# Predominance of eyes and surface information for face race categorization

**DOI:** 10.1038/s41598-021-81476-1

**Published:** 2021-01-21

**Authors:** Isabelle Bülthoff, Wonmo Jung, Regine G. M. Armann, Christian Wallraven

**Affiliations:** 1 Max Planck Institute for Biological Cybernetics, Max-Planck-Institut für biologische Kybernetik, Max-Planck-Ring 8, 72076 Tübingen, Germany; 2 Korea University, Seoul, South Korea

**Keywords:** Psychology, Human behaviour

## Abstract

Faces can be categorized in various ways, for example as male or female or as belonging to a specific biogeographic ancestry (race). Here we tested the importance of the main facial features for race perception. We exchanged inner facial features (eyes, mouth or nose), face contour (everything but those) or texture (surface information) between Asian and Caucasian faces. Features were exchanged one at a time, creating for each Asian/Caucasian face pair ten facial variations of the original face pair. German and Korean participants performed a race classification task on all faces presented in random order. The results show that eyes and texture are major determinants of perceived biogeographic ancestry for both groups of participants and for both face types. Inserting these features in a face of another race changed its perceived biogeographic ancestry. Contour, nose and mouth, in that order, had decreasing and much weaker influence on race perception for both participant groups. Exchanging those features did not induce a change of perceived biogeographic ancestry. In our study, all manipulated features were imbedded in natural looking faces, which were shown in an off-frontal view. Our findings confirm and extend previous studies investigating the importance of various facial features for race perception.

## Introduction

Faces offer a wealth of information which we use in everyday life not only to recognize familiar people, but also to classify unfamiliar ones fast and accurately according to their race, sex or age, among many other categories.

Facial information is processed at various levels of feature integration to achieve those tasks^1–4^. We can identify and describe various features in a face, for example we can report the color of the eyes or describe a nose to be aquiline. To discriminate between individuals, it is assumed that we use not only this type of featural information but also how those features relate to each other spatially (configural information)^5,6^. Furthermore, faces are also processed holistically, which means that the perception of a part of a face is always influenced by the other facial features, (see for example)^1,7–11^.

It is a subject of debate whether it is the higher expertise for own-race faces or the cultural background that might influence the importance of one or the other facial components for assessing face identity or ethnicity. In terms of expertise, it is known that observers show what is called the other-race effect for faces of an unfamiliar race. That is, they display reduced performance in a variety of face recognition tasks^12^ when other-race faces instead of same-race faces are tested. One main hypothesis is that they rely on facial features that have been optimized for discriminating between faces of their race of expertise (own-race faces), features that might not be best for discriminating between other-race faces. Experimental evidence reports that observers belonging to different racial background rely indeed on different facial features to describe faces^13^. This reflects the fact that faces of different races differ not only in terms of their facial features (e. g. shape of the eyes) but also in terms of variations of those feature^14–16^. Accordingly, some eye tracking studies have demonstrated that East-Asians as well as Westerners look differently at same- and other-race faces^17,18^.

Other studies have suggested that East-Asians and Westerners look at faces (and scenes) differently because of their different cultural backgrounds^19,20^. These findings are discussed in terms of stronger holistic viewing in Asian participants and more analytic strategies in Caucasian participants and/or reliance on different facial cues in both groups. Similarly, eye tracking studies have reported that Westerners distribute their gaze predominantly on eyes and mouth whereas East-Asians fixate more the center of the face (the nose), which might also correspond to a more holistic visual processing of East-Asians than Westerners^21,22^, but see in contrast^23^.

Race categorization has been often tested with participants of a single cultural background, leaving open the question whether their findings were valid for that cultural background only or were of more general value. Among the studies that have investigated the importance of facial components for race or sex perception^23–29^, most have used degraded face stimuli; that is, participants did not see complete or normal-looking faces. Example manipulations included restricting visibility to random parts of the face through small apertures (bubble method)^23,27,30^, or filtered faces (either low-pass or high-pass filtered)^28,31^. Lastly, faces were often shown frontally, a view that does not offer a good sight of the shape of the jaw line and of the nose, which might have led to an underestimation of the importance of that facial information^32^.

The present study addressed those potential problems first by using novel stimuli: we exchanged one of the facial features in a test face with the same feature from another race category, following the paradigm introduced first by Brown & Perrett^33^. The advantage of the method devised by Blanz and Vetter^34,35^, which we followed here, is that we were able to present faces with exchanged parts that remained natural-looking. Participants were not aware of the applied manipulation as the facial features were naturally embedded in whole faces contrarily to other studies using degraded face stimuli. With this approach, we ensured that the manipulated stimuli did not change how participants process faces or hindered holistic processing of the stimuli. In addition, our face manipulations preserved depth information as it worked in three dimensions; this allowed us to rotate all faces in 3D space to better assess the importance of the nose and contour for face race classification. Finally, we tested participants of two cultural backgrounds with the same stimuli and experimental paradigm (in Germany and Korea) to determine the importance of participants’ cultural background on ethnicity decisions.

In our study, we use the terms ‘race’ (used largely in the face categorization literature) and ‘biogeographic ancestry’ indifferently. Both terms refer here to the physical differences between faces with origin from two major world regions: Europe (Caucasian) and Asia (Asian). Importantly, the term ‘race’ does not refer to any concept of biological race as biological races do not exist for humans (see for example, the study of Cosmides and colleagues^36^ on this matter) but refers to how faces are grouped based on certain features.

## Methods

### Participants

We tested two groups of participants; the German group consisted of 48 Caucasian students from the University of Tübingen and employees of the Max Planck Institute for Biological Cybernetics (24 female, average age: 27). In South Korea, 48 students from Korea University (24 female, average age: 23) were tested. All participants had normal or corrected to normal vision and were naïve as to the purpose of the experiment. Half of each group performed the experiment with a monitor equipped with an eyetracker (eyetracker subgroup), whereas the monitor was not similarly equipped for the other participants’ half (no-tracker subgroup). All participants were paid volunteers and gave informed consent to participate in the study. The procedures were performed in accordance with the relevant guidelines and regulations and approved by the Ethical Review Board of the Max Planck Society (code number: 2016_02).

### Stimuli

**Figure 1:**
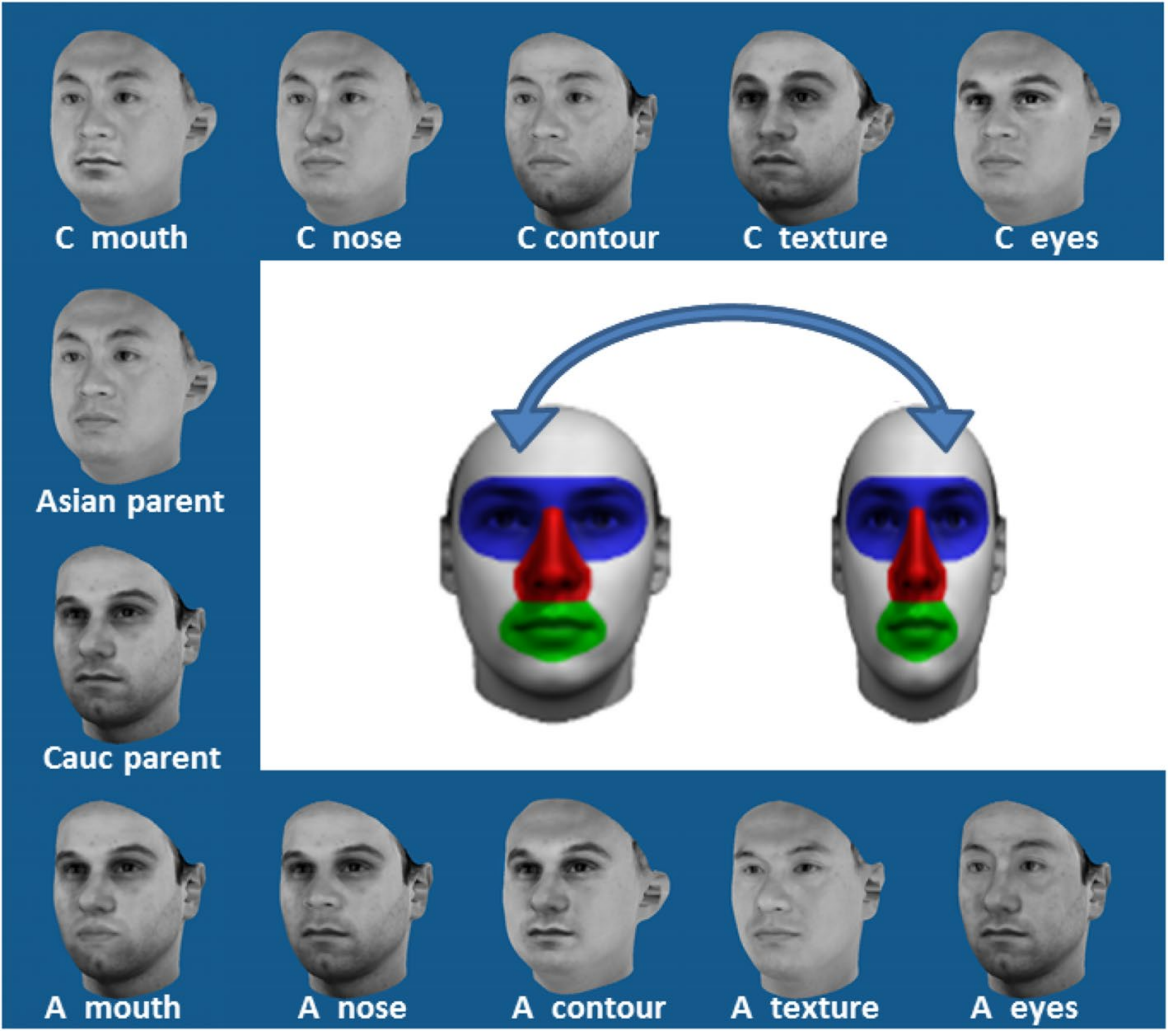
Example of face images derived from one Asian-Caucasian face pair (blue background images). Here only one of two face orientation is shown. A: Asian, C or Cauc: Caucasian. The top faces display the Asian parent faces with one Caucasian facial feature. The bottom faces display the Caucasian parent faces with one Asian facial feature. The central face pair on white background shows the facial regions (eyes: blue, nose: red, mouth; green, contour: grey) that were exchanged between faces. Texture (skin) was also exchanged between parent faces (as seen in A texture and C texture). The images were obtained using our in-house graphical softwares (face modeler (version 1.2.97) and the morphable model developed by Blanz and colleagues)^34,35^.

We selected 20 Asian and 20 Caucasian 3D laser-scans of faces, half being female, from our in-house face database^34,37^. Informed consent was obtained from the scanned people to publish their images in scientific publications. We paired each Asian face with a Caucasian face that shared the same sex, had approximately the same age and similar appearance (e.g. pairing two elongated faces) to create 20 Asian-Caucasian face pairs. In each face, three inner face regions were determined (eyes, nose and mouth) while a fourth one corresponded to everything else in the face (face contour). Those facial regions are depicted in the large central faces in Fig. 1. Using the morphable model developed by Blanz and colleagues^34,35^ and an in-house graphical interface (face modeler), we exchanged those facial regions between the faces of each pair automatically to create mixed-race faces. We also exchanged the facial texture (surface information) while keeping the original shape. All mixed-race faces had only one exchanged (other-race) facial feature. To reduce the visibility of potential color tone differences between original and exchanged facial parts, we used grayscale rendering of the faces. For obtaining stronger shape information about the face in the stimuli images, especially for the nose and jaw, the faces were rendered rotated 10° to the right along the vertical axis. In this view, eyes and mouth remain fully visible. The grey-scale faces were shown on a blue background. All face images were 550 × 550 pixels in size. For easy balancing of face orientation, in addition to the original stimuli, new images were created by flipping the images to show the faces turned to the other side. Thus each face pair generated a set of 24 images each (two parent faces and 10 mixed-race faces in two orientations). There are six types of faces: original faces (parents), eyes-exchanged faces, nose-exchanged faces, mouth-exchanged faces, contour-exchanged faces and texture-exchanged faces. The images of one set in one orientation are shown in Fig. 1. All Caucasian parent faces and their derived faces (Cauc parent, A nose, A contour, A texture A eyes in Fig. 1) are called faces of Caucasian origin, Conversely, all Asian parent faces and their derived faces (Asian parent, C nose, C contour, C texture C eyes in Fig. 1) are called faces of Asian origin.

### Design and procedure

Two (one male and one female) of the 20 sets were used exclusively during a training phase before starting the actual experiment. Each image subtended a visual angle of approximately 11° by 11° on a monitor (1920 X 1200 pixels) with a refresh rate of 60 Hz. Participants saw all faces one by one and classified them as Asian or Caucasian by pressing one of two response buttons of a button box. The assignment of response buttons was counterbalanced across participants. Face presentation order was randomized across participants for the no-tracker subgroup. Each participant of the eyetracker subgroup saw one of 2 random order presentations. Each image of the 18 test sets was presented twice resulting in 864 test trials (= 18 sets * 2 orientations * 12 conditions * 2 repetitions). The trials were divided in three blocks separated by self-timed breaks. The experiment was conducted using E-Prime software (Psychology Software Tools, Pittsburgh, PA) for the no-tracker subgroup and Tobii Studio software for the eyetracker subgroup. Each trial started with a fixation cross for 500 ms followed by a face for 1000 ms. A blank screen appeared next. Participants could answer as soon as the face appeared on the screen. Participants were told to be as fast and as accurate as possible. Independent variables were ethnicity choice (all participants) and response time (no-tracker subgroup only).

### Statistical analysis

Separate 2 X 6 repeated measures ANOVAs were carried out for each participant group. Parent race (Asian, Caucasian) and face type (original, exchanged mouth, exchanged nose, exchanged contour, exchanged texture, exchanged eyes) were within-subject factors. An alpha of 0.05 was used for statistical significance. When the Mauchley’s test of sphericity was significant, a Greenhouse–Geisser correction was applied. Effect sizes such as using partial eta squared values (η_p_^2^) and Cohen’s *d* are reported for *F*-tests and two-tailed *t*-tests, respectively. Bonferroni corrections were applied for multiple comparisons.

## Results

### Race choices

**Figure 2:**
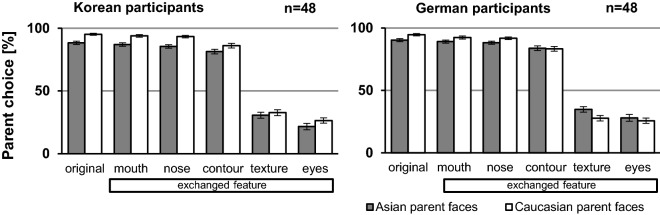
Ethnicity classification as percentage of parent race for faces without (original) or with featural modifications (mouth, nose, contour, texture or eyes). Error bars denote standard error of the means.

The graphs in Fig. 2 show how often participants in each country classified each face type according to the ethnicity of its parent face (% parent choice). For the Korean group, the main effects of parent race and face type were significant (parent race: *F*(1, 47) = 4.45, *p* = 0.040, η_p_^2^ = 0.09; face type: *F*(1.50, 70.31) = 907.02 *p* < 0.001, η_p_^2^ = 0.95) whereas the interaction between those factors was not (*F*(2.06, 96.74) = 2.69 *p* = 0.072, η_p_^2^ = 0.05). The result shows that faces of Caucasian origin were classified more often as Caucasian than their Asian counterparts were as Asian. Furthermore, face type affected classification as clearly visible on the graph, this effect on classification was similar for Asian and Caucasian faces.

For the German group, the interaction between both factors was significant (*F*(1.93, 90.62) = 11.58, *p* < 0.001, η_p_^2^ = 0.198) indicating that the facial alterations affected the classification of faces derived from Asian and Caucasian parents differently depending on which facial feature was exchanged. On the graph, it is visible that for the original faces and for mouth, nose and contour face types, Caucasian faces were categorized as Caucasian more often than their Asian counterparts as Asian, whereas it was inversed for texture and eyes versions. There was also a significant effect of face type (*F*(1.63, 76.46) = 771.24, *p* < 0.001, η_p_^2^ = 0.943), exchanged eyes, for example, influenced classification far more than an exchanged nose, as is clearly visible on the graph. There was no main effect of face race (*F*(1, 47) = 0.01 *p* = 0.925, η_p_^2^ = 0.00), which indicates that, overall, faces of Caucasian or Asian origin were race-classified similarly.

Do all face modifications significantly alter race perception? To answer this question, we ran paired t-tests comparing responses to parent stimuli with responses to their modified versions (Table 1). For Korean participants, modification of any feature significantly lowered race classification compared to the parent faces except for the exchanged mouth face type. For German participants, modification of any feature lowered significantly race classification compared to the parent faces except when the mouth in Asian parents was swapped for a Caucasian one. In sum, among all investigated facial modifications, exchanging the mouth affected the least the race perception of the face it was introduced in.

**Table 1:** Paired t-tests comparing categorization responses for the original parent faces to responses for their face variations.

Korean participants			German participants		
Comparison	*t*(47) =	*p* = *d *=	Comparison	*t*(47) =	*p* = *d* =
a_original—a_cmouth	2.52	.015 0.365	a_original—a_cmouth	1.88	.066 0.271
a_original—a_cnose	5.28	** < .001** 0.761	a_original—a_cnose	3.14	**.003** 0.454
a_original—a_ccontour	6.71	**< .001** 0.969	a_original—a_ccontour	5.26	**< .001** 0.758
a_original—a_ctexture	30.93	**< .001** 4.465	a_original—a_ctexture	28.41	**< .001** 4.102
a_original—a_ceyes	28.87	** < .001** 4.167	a_original—a_ceyes	26.23	**< .001** 3.787
c_original—c_amouth	2.47	.017 0.357	c_original—c_amouth	3.20	**.002** 0.461
c_original—c_anose	3.07	**.004** 0.443	c_original—c_anose	3.60	**.001** 0.520
c_original—c_acontour	7.17	< .001 0.872	c_original -c_acontour	8.68	**< .001** 1.252
c_original—c_atexture	27.51	**< .001** 3.004	c_original—c_atexture	33.08	**< .001** 4.773
c_original—c_aeyes	33.22	**< .001** 3.577	c_original—c_aeyes	31.41	**< .001** 4.534

Significant p-values after Bonferroni correction for multiple significance tests (n = 10) are shown in bold.

a_original: Asian parent, a_cmouth: Asian face with Caucasian mouth, a_cnose: Asian face with Caucasian nose, a_ccontour: Asian face with Caucasian contour, a_ctexture: Asian face with Caucasian texture, a_ceyes: Asian face with Caucasian eyes, c_original: Caucasian parent, c_amouth: Caucasian face with Asian mouth, c_anose: Caucasian face with Asian nose, c_acontour: Caucasian face with Asian contour, c_atexture Caucasian face with Asian testure, c_aeyes: Caucasian face with Asian eyes.

Exchanging eyes or texture in a face elicited a change in race perception for that face. How strongly are these modified faces perceived as belonging to the other race category? The values in Table 2 show ethnicity classification as percentage of the race of the exchanged features and the categorization values obtained for the parent faces of the same race. On the one hand, paired t-tests comparing those values confirm that parent Asian (Caucasian) faces were more often classified in their race category than faces with exchanged Asian (Caucasian) eyes or texture for both groups (all *t*s(47) > 8.00, all *p*s ≤ 0.001, all *d*s > 1.282). On the other hand, one-sample t-tests comparing race classification of those face types (eyes, texture) to ambiguous classification (50%) revealed that those modifications did not result in racially ambiguous-looking faces (all *t*s(47) ≥ 6.89, all *p*s ≤ 0.001, all *d*s > 0.994).

**Table 2:** Mean ethnicity choices for the race of the exchanged feature (eyes or texture) given in percent and calculated for each participant group.

Face type	Korean participants	German participants
	Mean ± SEM	Mean ± SEM
Asian parent	88 ± 1	90 ± 1
Exchanged Asian eyes	74 ± 2	74 ± 2
Exchanged Asian texture	67 ± 2	72 ± 2
Caucasian parent	95 ± 1	95 ± 1
Exchanged Caucasian eyes	78 ± 2	72 ± 3
Exchanged Caucasian texture	69 ± 2	65 ± 2

The categorization values for the parent faces are given for comparison.

SEM: standard error of the mean.

Because of the reported differences in how East-Asian and Westerner look at faces^17,38^, we investigated whether the Korean group would give more importance to the nose than the German group and also checked whether it was the reverse for the importance given to the eyes (Table 3). Independent sample t-tests revealed that exchanging nose or eyes in a face similarly modified race perception in both groups (all *t*s(190) ≤ 1.172, all *p*s ≥ 0.243).

**Table 3:** Mean ethnicity choices for the parent race in percent and standard error of the mean (SEM) calculated for each participant group for faces with exchanged nose or eyes.

Face type	Korean participants	German participants
	Mean ± SEM	Mean ± SEM
Face with exchanged nose	88 ± 1	90 ± 1
Face with exchanged eyes	24 ± 2	27 ± 2

### Response times

**Figure 3:**
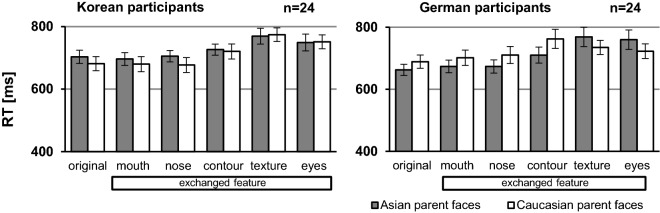
Response times for faces without (original) or with featural modifications (mouth, nose, contour, texture and eyes). Error bars denote standard error of the means.

Response times of participants performing the task on a monitor equipped with an eyetracker were not recorded. Because of the mixed-race face trials, response times of the remaining 24 Korean and 24 German participants were calculated over all trials. The graphs in Fig. 3 show that both groups tended to respond fastest to the original parent faces and also faster to other-race faces than to same-race faces, but not when eyes or texture changes were involved; for those conditions response times differences were eliminated or inversed.

The interaction between both factors was significant for the German group (*F*(1.38, 31.84) = 6.58, *p* = 0.009, η_p_^2^ = 0. 222), indicating that the facial alterations affected own-race and other-race faces differently. In this group, other-race faces were classified slightly faster than own-race faces, except when the eyes or the texture was exchanged. For the Korean group, we observe the same pattern, but the interaction did not attain significance (*F*(1.58, 36.29) = 1.263, *p* = 0.288, η_p_^2^ = 0.052). For both groups, there was also a significant effect of face type (Germans: *F*(1.88, 43.28) = 35.93, *p* < 0.001, η_p_^2^ = 0.610, Koreans: *F*(2.95, 67.79) = 39.61, *p* < 0.001, η_p_^2^ = 0.633) and no main effect of face race (Germans: *F*(1, 23) = 3.28, *p* = 0.083, ηp2 = 0.125; Koreans: *F*(1, 23) = 3.54, *p* = 0.073, η_p_^2^ = 0.133) .

It is noteworthy that stimuli belonging to face types that were perceived as own-race faces by the participants, (see Fig. 2) were always responded to more slowly than stimuli belonging to face types perceived as other-race faces (Fig. 3). In more detail, Fig. 2 reveals that Germans perceived the face types Caucasian parent, Caucasian face with Asian mouth, Caucasian face with Asian nose, and Caucasian face with Asian contour to be Caucasian, as well as Asian face with Caucasian texture and Asian face with Caucasian eyes. The same in reverse appears in the Korean group. We reanalyzed the data after changing the face type eyes and texture to their perceived race category to investigate whether perceived face race (own race, other race) influenced response times differently. For the German group, the new ANOVA revealed a significant effect of face race (*F*(1,23) = 7.12, *p* = 0.014, η_p_^2^ = 0.236; response times to perceived own-race faces *M* = 732 ms ± SEM 27, response times to perceived other-race faces *M* = 696 ms ± SEM 2). Thus, after that change of race affiliation, perceived other-race faces were answered to significantly faster than perceived own-race faces. Face type remained a significant main effect (*F*(1.882, 43.282 = 35.93, *p* ≤ 0.001, η_p_^2^ = 0.610) and there was no more significant interaction (*F*(1.384, 31.839) = 1.51, *p* = 0.193, η_p_^2^ = 0.061). We also reanalyzed the data for the Korean groups: although perceived other-race faces were responded to faster than perceived own-race faces (response times to perceived own-race faces *M* = 726 ms ± SEM 19, response times to perceived other-race faces *M* = 713 ms ± SEM 24), the response time differences between perceived own- and other-race faces was too small for obtaining any significant effect of perceived face race (*F*(1,23) = 1.59, *p* = 0.220, η_p_^2^ = 0.065). Face type remained a significant main effect (*F*(2.947,67.786 = 39.61, *p* ≤ 0.001, η_p_^2^ = 0.633) with no significant interaction (*F*(3.48, 80.09) = 2.21, *p* = 0.084, η_p_^2^ = 0.088).

We also compared response times when classifying own-race and other-race parent (original) faces alone. Here, participants in both groups classified other-race faces faster than own-race faces (Table 4), but paired t-tests revealed that this trend did not reach significance (Germans: *t*(23) = 1.96, *p* = 0.062, *d* = 0.401, Koreans: *t*(23) = 1.65, *p* = 0.113, *d* = 0.145).

**Table 4:** Mean response times to the parent faces, ± standard error of the mean.

Group	Asian parents	Caucasian parents
Koreans	703 ms ± 21 ms	681 ms ± 23 ms
Germans	663 ms ± 18 ms	689 ms ± 22 ms

Do all face modifications alter significantly response times? We ran paired t-tests comparing response times to parents and to their modified versions (Table 5). For Korean participants, response times to other-race (Caucasian) faces slowed down significantly when contour, eyes and texture were exchanged whereas changing mouth and nose did not. For same-race (Asian) faces, only faces with a Caucasian texture were responded to significantly more slowly than the original faces. For German participants, response times to other-race (Asian) faces were slowed down when contour, texture and eyes were exchanged whereas mouth and nose did not modify response times significantly. Response times to same-race (Caucasian) faces slowed down significantly when contour or texture was exchanged. For both groups, the analyses suggest that strong race indicators (contour, eyes and texture) affect response times whereas weak race indicators (mouth and nose) do not.

**Table 5:** Paired t-tests comparing response times for original parent faces to response times for their face variations for Korean and German participants.

Korean participants			German participants		
Comparison	*t*(23) =	*p * = *d* =	Comparison	*t*(23) =	*p * = *d * =
a_original—a_cmouth	1.14	.266 0.231	a_original—a_cmouth	1.66	.110 0.340
a_original—a_cnose	0.22	.831 0.044	a_original—a_cnose	1.43	.167 0.293
a_original—a_ccontour	2.43	.024 0.495	a_original—a_ccontour	4.72	**< .001** 0.893
a_original—a_ctexture	3.71	**.001** 0.758	a_original—a_ctexture	5.42	**< .001** 1.106
a_original—a_ceyes	2.59	.016 0.529	a_original—a_ceyes	4.95	**< .001** 1.011
c_original—c_amouth	0.23	.819 0.047	c_original—c_amouth	1.73	.097 0.352
c_original—c_anose	0.62	.541 0.127	c_original—c_anose	2.78	.011 0.565
c_original—c_acontour	5.68	**< .001** 1.164	c_original—c_acontour	5.65	**< .001** 1.153
c_original—c_atexture	7.63	**< .001** 1.554	c_original—c_atexture	2.41	**.002** 0.719
c_original—c_aeyes	7.62	**< .001** 1.555	c_original—c_aeyes	2.81	.010 0.574

Significant p-values after Bonferroni correction for multiple significance tests (n = 10) are shown in bold. For more details, see Table 1.

### Race ambiguity and response times

**Figure 4:**
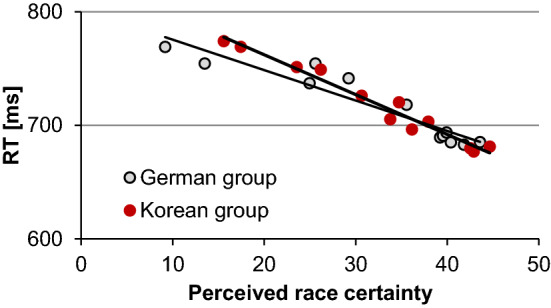
Relation between perceived race certainty calculated for each face types of both ethnicities together and response times for each participant group. Black lines: linear fits.

For participants from whom we have response time and ethnicity choice data, we calculated a ‘race certainty’ value for each face type. This was determined as the absolute value of the difference between the classification value and 50% as an index of how perceptually-ambiguous a face is in terms of its race. We then determined whether these values correlated (negatively) with response times as racially more ambiguous faces might require more time for classification (Fig. 4). Race certainty was indeed strongly negatively correlated with response time (Pearson correlation for Koreans: *r*(10) = −  0.98, *p* < 0.001; for Germans: *r*(10) = − 0.87, *p* < 0.001.

## Discussion

Our results show clearly that participants in Germany and Korea race-classified most faces with exchanged mouth, nose or contour according to the ethnicity of the *original* parents, whereas exchanging eyes or facial texture had a drastic effect as participants changed their classification to answer in accordance to the race of the exchanged feature. These findings demonstrate unambiguously that eyes along with face texture are the two most potent features in giving a face its race.

Concerning the eyes, our results demonstrate that the importance of the eyes as race indicator is independent of the race of the parent faces and of the cultural background of the observers. In addition, our findings extend previous studies revealing the important role of the eyes for race perception^23,27,29^ by demonstrating that eyes placed in a face of a different ethnicity are sufficient for determining the perceived race of the whole face.

So far, the importance of face texture (compared to shape information) has been only investigated for the recognition of own- and other-race faces^39^. The authors of that study reported that its importance depended on the cultural background of the participants and the quality of the faces (same-race, other-race). In our study, we used grey-shaded pictures. The lack of color might have reduced the importance of the texture, and furthermore the faces were turned to the side, which facilitates the use of shape for performing the task. Nevertheless, texture revealed itself as a major component for race perception. In view of the grey-shaded pictures, skin tone cannot have been the crucial element for determining race perception. As the influence of the mouth and the nose has been tested separately and shown to be low, eyes and eyebrows might have been the crucial elements for race perception in exchanged-texture stimuli. In our paradigm, eyebrows were not investigated separately from the eyes. Sadr, Jarudi, and Sinha have previously demonstrated the importance of eyebrows for face identification^40^, but their importance for race categorization remains to be investigated.

Our results also demonstrate that the importance of the mouth and the nose for race perception remained low despite nose shape being more clearly visible given the rotated face presentation. In faces with an exchanged contour, the inner features (eyes, nose and mouth) are kept, whereas the whole surrounding (cheeks, chin, jaw and forehead) are exchanged (Fig. 1). With the off-frontal orientation, the shape of the jaw and the chin are rendered more visibly. Despite the comparatively large size of the exchanged area, its influence on race perception was not much stronger than exchanging the mouth or the nose alone. This finding stresses the importance given to the inner facial features for race assessment.

Further analyses showed that the influence of nose and eyes on race perception was similar for German and Korean participants despite the reports that Asian participants concentrate their gaze in a more holistic fashion, that is toward the center of the face (the nose), while Westerners would distribute their gaze onto the eyes and the mouth in a triangular fashion^21,22^. Our study reveals that despite these different gaze behaviors, participants of East-Asian and Western cultures accord the same high diagnostic importance to the eyes and low importance to the nose for race assessment.

Previous studies have evidenced that there is an other-race classification advantage (ORCA); other-race faces are race-categorized faster than own-race faces^29,41–43^. In accordance with this concept, participants in our study also tended to respond faster (although not always significantly) to faces perceived to belong to another ethnicity than their own. We suppose that the rather weak ORCA found in our study is due to our paradigm that showed many “mixed-race” faces and therefore participants were more hesitant about how to race-classify all face stimuli than when only original faces are shown.

Importantly, response times correlated clearly negatively with face race ambiguity for both groups of participants. Furthermore, all investigated exchanged facial features—except for the mouth–, significantly influenced how often a face was perceived as Asian or Caucasian and facial features influencing most race perception also slowed down participants’ responses even further. Together our findings suggest that all facial features are taken into account when assessing race: although the eyes and face texture are by far the most potent race indicators, our analyses confirm that exchanging the eyes (or the texture) in a face for eyes (or texture) of another race does not change race categorization of the resulting faces ‘completely’. In other words, the remaining parent features in the manipulated faces are not ignored and categorization values for the face type eyes and texture never equaled those obtained with the original parents.

Note that the present study did not investigate the role of configuration for race categorization. In their study, Bentin and his colleagues^28,29^ manipulated spatial frequency scales of face stimuli, but did not manipulate facial features. They reported that race determination was based predominantly on global and configural information. It would be interesting to combine spatial frequency and facial feature manipulations to obtain a more complete picture of what makes the race of a face^2^.

## Data Availability

None of the materials, but the data for the experiments reported here are available upon request.
